# An Efficient Three-Dimensional Convolutional Neural Network for Inferring Physical Interaction Force from Video

**DOI:** 10.3390/s19163579

**Published:** 2019-08-17

**Authors:** Dongyi Kim, Hyeon Cho, Hochul Shin, Soo-Chul Lim, Wonjun Hwang

**Affiliations:** 1 Department of Software and Computer Engineering, Ajou University, 206 Worldcup-ro, Yeongtong-gu, Suwon 16499, Korea; 2 Department of Mechanical, Robotics and Energy Engineering, Dongguk University, 30, Pildong-ro 1gil, Jung-gu, Seoul 04620, Korea

**Keywords:** deep learning, force estimation, interaction force, convolutional neural network

## Abstract

Interaction forces are traditionally predicted by a contact type haptic sensor. In this paper, we propose a novel and practical method for inferring the interaction forces between two objects based only on video data—one of the non-contact type camera sensors—without the use of common haptic sensors. In detail, we could predict the interaction force by observing the texture changes of the target object by an external force. For this purpose, our hypothesis is that a three-dimensional (3D) convolutional neural network (CNN) can be made to predict the physical interaction forces from video images. In this paper, we proposed a bottleneck-based 3D depthwise separable CNN architecture where the video is disentangled into spatial and temporal information. By applying the basic depthwise convolution concept to each video frame, spatial information can be efficiently learned; for temporal information, the 3D pointwise convolution can be used to learn the linear combination among sequential frames. To validate and train the proposed model, we collected large quantities of datasets, which are video clips of the physical interactions between two objects under different conditions (illumination and angle variations) and the corresponding interaction forces measured by the haptic sensor (as the ground truth). Our experimental results confirmed our hypothesis; when compared with previous models, the proposed model was more accurate and efficient, and although its model size was 10 times smaller, the 3D convolutional neural network architecture exhibited better accuracy. The experiments demonstrate that the proposed model remains robust under different conditions and can successfully estimate the interaction force between objects.

## 1. Introduction

Humans possess the inherent capacity to sense the surroundings for their survival and safety. Among man’s five senses—hearing, touch, smell, taste, and sight—the latter receives relatively large amounts of information and transmits these to the brain. Moreover, human senses complement each other and seemingly learn from experiences; when any of these senses do not function properly, the other senses compensate for the deficiency by using information from previous experiences. Consider the following. A person who has previously seen and touched hard and soft objects can anticipate how another object feels even before touching it; based on experience, a person can also infer that certain objects are hot or cold even before actually touching them. In light of the foregoing, this study focused on the capacity of the sense of sight to perceive visual information; furthermore, the relationship between touch and sight was examined, and achieving tactile sensing through vision based on a deep learning framework was explored.

Over several years, haptic sensing in robotics has been a topic of considerable interest [1,2,3]. Haptic sensing is measured by means of a value such as that of an interaction force between an object and instrument. In grasping and manipulating situations, the information pertaining to such a force is useful so that it can be successfully employed [4]. Well-known sensing methods are implemented through electromagnetic induction [5] to determine the small power for an interaction force and the torque sensor [6] for a large interaction force. Meanwhile, the sense of sight can be easily implemented using a camera device. In this regard, over the last two decades, several visual applications have been proposed from the hand-craft feature [7] to deep learning [8], in order to recognize visual information. In the case of video-based recognition, the convolutional neural network (CNN) + long short-term memory (LSTM)-based method [9] has recently exhibited impressive performance. The method for sharing visual information and tactile sensing has also been studied from the perspective of neuroscience [10]. The article in [11] reports that the human brain uses shared models of objects across multiple sensor modalities. In this respect, we can infer that knowledge can be transferred from tactile sensing to visual sensing. In particular, Tiest and Kappers [12] have examined the human brain’s capability to seemingly perceive haptic stimulus as they observed hands interacting with objects in certain configurations. Based on [12], the approach to simulate the interaction force estimation by using only video data was investigated. Note that this is similar to the human capability of perceiving the interaction force from images based on experience. In this study, this capability was mimicked using a deep learning method. The proposed method can be trained to identify visual variations of object shapes associated with external forces so that it can infer the interaction force from a video without the use of a haptic device.

**Figure 1:**
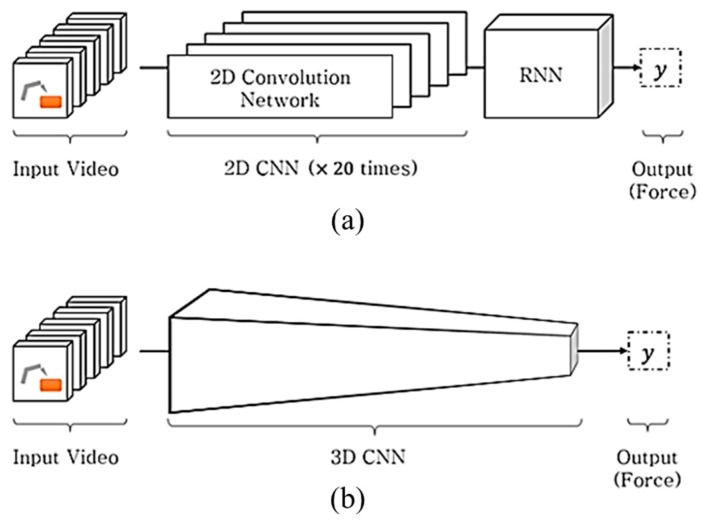
(**a**) Heterogenous network structure in the Convolutional Neural Network (CNN) + Long Short-Term Memory (LSTM) method and (**b**) homogeneous network structure in 3D CNN method. Both methods are proposed for predicting the interaction force from the input video.

To achieve this objective, the basic deep learning network was subjected to an ablation test [13], where a simple recurrent neural network (RNN) is implemented with limited training; the potential of this approach was demonstrated using the proposed method. In this paper, we proposed the light-weighted three-dimensional (3D) CNN-based force estimation method. In the CNN + RNN [9]-based method shown in Figure 1a, the CNN analyzes the spatial information, whereas the RNN concurrently manages the time series information. On the other hand, as shown in Figure 1b, the 3D CNN-based method [14] is composed of a homogenous network that analyzes the spatial and temporal information in a single framework, which is advantageous for weight parameter reduction. Although deep learning-based methods have adequately performed in computer vision applications, the computational complexity involved is a burden in real applications. Accordingly, in some applications (e.g., drone-based applications [15]), the hand-crafted features, which have a lower computational complexity than the deep learning-based methods, are preferred. To achieve the objective of this study, we collected 387,473 sequential images from an automatic training database collection system; the images were captured in various situations such as three different illuminations and four pose angles. To process these data, the bottleneck-based 3D depthwise separable convolution (DSC) neural network was proposed; the network functions as a deep learning model with small weight parameters and has been found to exhibit superior prediction accuracy in inferring interaction forces from images.

The rest of this paper is organized as follows. Section 2, describes the related works. In Section 3, the basic configuration of the proposed 3D CNN-based model architecture is elaborated. In Section 4, the database collection method, experimental results, and discussion are presented. Finally, the conclusions drawn are summarized in Section 5.

## 2. Related Works

Studies on sensing interaction force without using a torque or tactile sensor have been previously performed. The study on humanoids in [16] proposed a method for reconstructing interaction forces and localizing the contact point in the static hypothesis based on joint torque. In [17], a sensorless gripping estimation method was suggested for forceps using both encoder and joint torque sensing; the method utilized the characteristics of elongated cable-driven surgical instruments. From the perspective of camera-based prediction methods, Aviles et al. [18] demonstrated the estimation of an applied force using a stereo camera for a surgical robotic system. The heart surface was reconstructed with a 3D model using stereo-pair images, then the supervised learning method was trained to determine the applied force from the deformable 3D model. In [19], the study took into consideration the physical quantities generated from 3D modeling and used these to predict the force between a human body and a real-world object. With known geometrical and physical properties, Pham et al. [20] demonstrated the estimation of the contact force in a hand–object interaction; the estimate was solely based on the visual input provided by a single RGB-D camera.

There have been early attempts to determine a methodology to measure the external force based only on video inputs [13,21]. In [13], images were collected, and a method was designed to determine the corresponding forces of different materials in the images. This proved that images could be employed for inferring interaction forces. However, it was designed using a simple RNN as a shallow network, and it could not train the network model using different materials simultaneously. Compared with [13], more videos and interaction forces were collected in the present study, and the proposed method was based on a 3D CNN instead of the RNN. According to this protocol, large sizes of databases can be learned and evaluated at once. This study further aimed to design a deep learning network with small weight parameters that could achieve good performance without degradation. The latter aim is achievable, as indicated by the study in [21], which reported that the interaction forces among rigid objects using electrical currents could be predicted using the well-known CNN + LSTM method as the baseline architecture [22]. Note that in the present work, the baseline network architecture was a 3D CNN-based method and not a CNN + LSTM method.

Accordingly, a neural network model with small weight parameters that can predict the interaction between an instrument and target object without using physical haptic sensors was proposed in this paper. The main contributions of this study are summarized as follows. (1) A more accurate and lightweight end-to-end neural network structure that can predict the physical quantities between an instrument and object was developed. (2) An efficient 3D convolutional network design method by extending the lightweight technique applied to the existing 2D convolutional network was formulated. (3) A large dataset that could learn and evaluate inferences regarding the interaction among objects was collected.

## 3. Proposed 3D CNN-Based Method

In this section, the concept of a 2D depthwise separable convolution is briefly described [23,24]. This convolution was modified to reduce the parameter sizes of the 3D convolutional neural network for inferring the interaction forces from a video input.

### 3.1. Two-Dimensional Depthwise Separable Convolution (DSC) Neural Network

**Figure 2:**
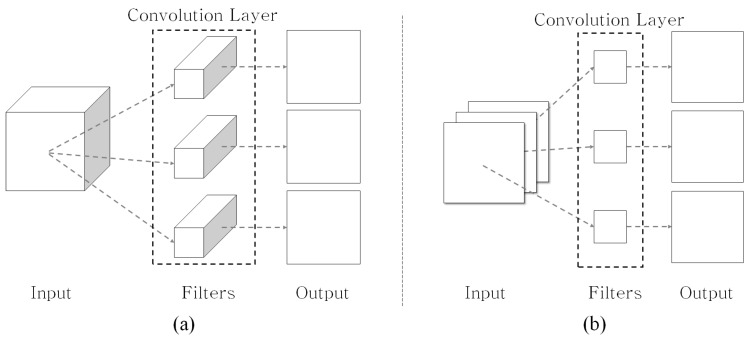
(**a**) Traditional 2D CNN and (**b**) 2D depthwise CNN concepts.

Traditional 2D CNN kernels were trained using all of the input channels to represent the input’s spatial information. The visual feature with height (h), width (w), and channel (c) is represented by $X\in {ℜ}^{h\times w\times c}$, and the convolutional filter, $F\in {ℜ}^{k\times k\times c}$, with a k×k kernel, produces the corresponding output, $Y\in {ℜ}^{h\times w}$, by the convolutional operation. The total number of outputs depends on the number of filters used. In several deep CNN methods [25,26], the higher layers utilize several filters for analyzing the high-level visual information, therefore, the total size of the deep learning model is also increased. To resolve this problem, the squeeze network architecture [27] was introduced. Recently, 2D depthwise CNN (DWC) methods [23,28] have been proposed. In these methods, the DWC filters, $F_{DWC}\in {ℜ}^{k\times k}$, are individually trained according to their corresponding channels, as illustrated in Figure 2b. Moreover, unlike a traditional 2D CNN, when the channel of the input increases, only the corresponding number of filters increases, and the total size does not. Consequently, the DWC-based methods can efficiently reduce the computational complexity of the traditional CNN method.

**Figure 3:**
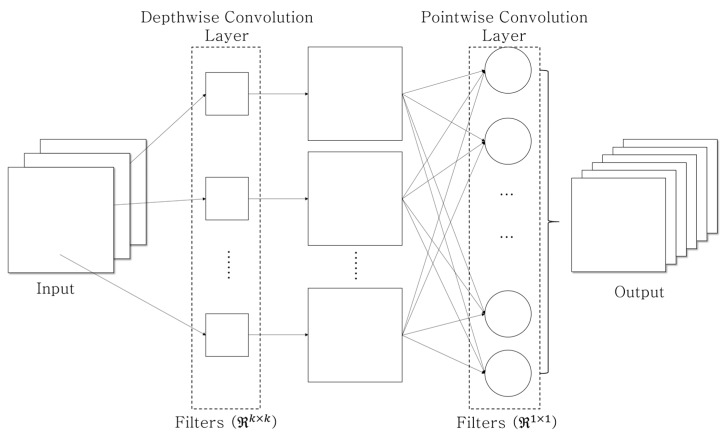
Depthwise separable convolution concept consists of the depthwise convolution (e.g., k=3) and the pointwise convolution.

On the other hand, depthwise convolutional filters are trained independently for each channel and do not successfully utilize the spatial information among channels; consequently, this can lead to performance degradation. In [28], the depthwise separable convolution (DSC) filter, which combines the DWC filter with the pointwise convolution (PWC) filter, $F_{PWC}\in {ℜ}^{1\times 1}$, for learning the correlation among the channels in the layer ends, was proposed. As shown in Figure 3, the DSC-based methods [23,24] could overcome the DWC’s deficiency (i.e., the absence of learning the information among channels) and simultaneously reduce the computational complexity involved in the deep learning network. In the end, the DSC-based approach is similar to decomposing the existing 2D convolutional layer into two separate procedures: the DWC layer and PWC layer.

### 3.2. Three-Dimensional Convolutional Neural Network

**Figure 4:**
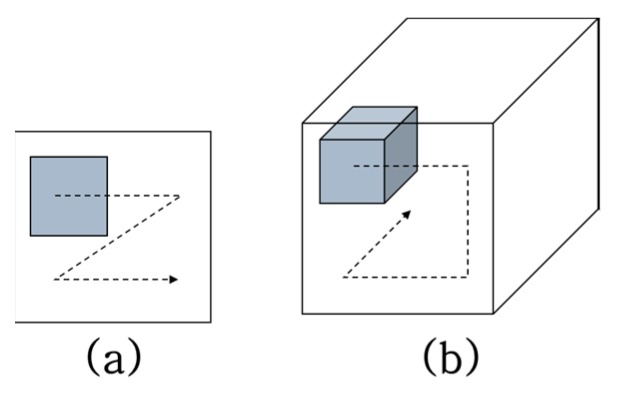
Concepts of (**a**) 2D convolution and (**b**) 3D convolution.

The 3D convolutional neural network is an extended version of the 2D CNN, which has another temporal direction, as shown in Figure 4. In extracting the sequential images from the video, the 3D CNN could also learn the spatiotemporal information from them. In this respect, with the 3D CNN, it is possible to learn the correlation of temporal changes between adjacent frames without employing additional temporal learning methods such as the recurrent neural network (RNN) [13]. Specifically, compared with the CNN + LSTM [9], the 3D CNN works successfully in the field of action recognition where the temporal information has a pivotal function in classifying human action. However, the 3D CNN has an inherent disadvantage: it has high computational complexity and excessive memory usage, which are major burdens in using the 3D CNN for several applications.

The 3D CNN parameter is calculated by the following equation:

(1) $$P_{3D}=n\times c\times d\times \left(k\times k+1\right)$$

where n is the number of filters; k is the spatial size of the convolutional kernel; d is the number of temporal images; and c is the number of channels. Therefore, similar to the 2D CNN, the 3D CNN is burdened with an increase in parameter size as well as computational complexity according to the number of input channels. The 3D CNN has an additional parameter, d, which is related to the number of adjacent images and learns the independent spatial information from each sequential image. In this respect, the computational complexity is further increased according to the number of sequential images used as input.

### 3.3. Proposed 3D Depthwise Separable Convolution Neural Network

**Figure 5:**
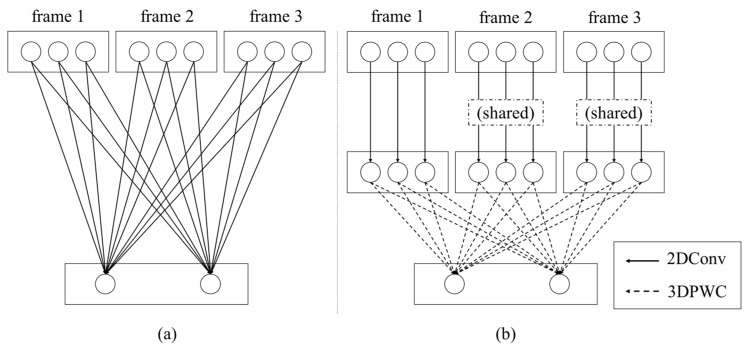
Concepts of (**a**) 3D CNN and (**b**) proposed 3D depthwise separable CNN. The rectangles and circles represent the video frames and their corresponding channels, respectively. For example, (**a**) is a video with three frames as the input with each frame consisting of three channels. The output of 3D CNN is an output with two channels because the 3D CNN has two filters. (**b**) In the proposed method, three channels of a frame are independently applied with three corresponding 2D convolution filters, then the final output with two channels is made by two 3D PWC filters.

In this section, a novel 3D depthwise separable CNN (DSC) is proposed to efficiently infer the interaction force only from a video clip. A similar approach [29] was applied to 3D classification using 3×3×3 filters. However, in this paper, to infer the haptic force from images, we disentangled the video into spatial information and temporal information, which it thereafter learnt independently and sequentially. Specifically, the following were performed. (1) Spatial feature extraction: the 2D depthwise convolution was applied to each frame of the input video; that is, the process of learning the spatial information independent of the channel was applied to each frame. (2) Temporal feature extraction: the 3D pointwise convolution was applied to learn the linear combination among the channels of adjacent frames. Figure 5 illustrates the comparison between the traditional 3D CNN and the proposed 3D DSC method. Compared with the traditional 3D CNN (Figure 5a), the proposed 3D depthwise separable CNN method first extracts the spatial feature information based on the 2D DWC method in the first sequential image from the video clip; the same 2D DWC filters are used for the other sequential images. Thus, in Figure 5b, shared marks were found on frames 2 and 3. Note that shared weight parameters were used in the proposed method, and the number of these parameters was not significantly increased when compared with the traditional 3D CNN. In the end procedure of the proposed method, the 3D PWC filters were employed to extract the temporal feature information.

As derived in Equation (1), the total number of parameters in the 3D CNN could be calculated. The sizes of the weight parameters were also derived, as listed in Table 1. Consequently, it was observed that the increase in computational burden caused by increasing the number of filters (n) and channels (c) was relatively less in the proposed architecture. Note that the number of filters and channels was directly related to the accuracy of the deep learning method and total size of the weight parameters.

**Figure 6:**
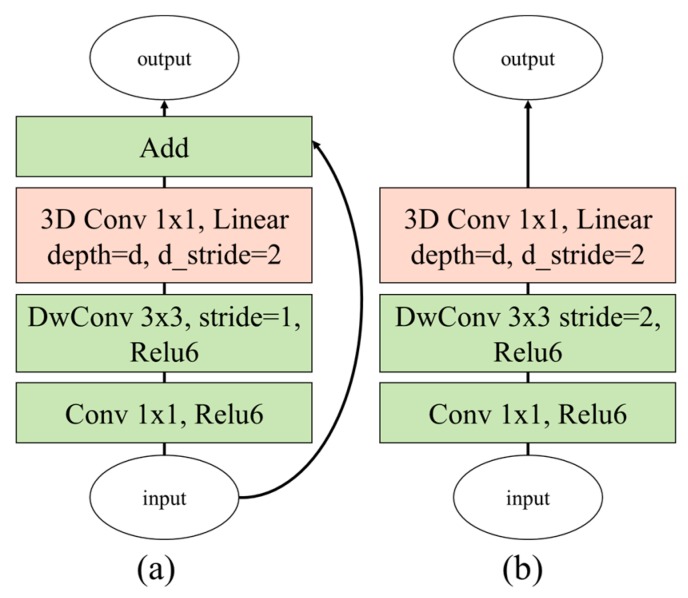
Proposed bottleneck 3D modules based on (**a**) inverted residual block and (**b**) linear block (DwConv: depthwise convolutional filter).

The proposed 3D depthwise separable convolution (DSC) filter was directly inspired by MobileNet V2 [30]; the proposed method was extended from the 2D image-based filter to the 3D frame-based filter. Figure 6 illustrates the extended bottleneck 3D modules for the inverted residual and basic linear block-based modules. The first layer of the proposed bottleneck 3D module for increasing the number of channels is the PWC, because even with the same parameter size as the conventional 3D CNN method, the proposed method can utilize more channels for inferring the interaction forces. The second layer is the depthwise convolutional filter with a 3 × 3 kernel; the 3D PWC is used in the last layer for learning the temporal information. As shown in Figure 6a, the inverted residual block-based modules at the beginning of the proposed network architecture are stacked several times to analyze the spatial and temporal images in detail. Thereafter, as shown in Figure 6b, the linear block-based modules are stacked successively to convert the temporal information to salient information to infer the interaction forces. The details of the network architecture of the proposed method are summarized in Table 2.

## 4. Database and Evaluation Protocol for Interaction Force Estimation

This section describes the database collection process for estimating the interaction force and the evaluation protocols to be used in performing comparisons with related works. Compared with [13], the new database collection method in this study differed as follows: (1) an automatic collection device was designed, (2) the number of target objects was increased, and (3) the collection environment conditions such as the moving direction and light changes could be varied.

### 4.1. Automatic Dataset Collection System

**Figure 7:**
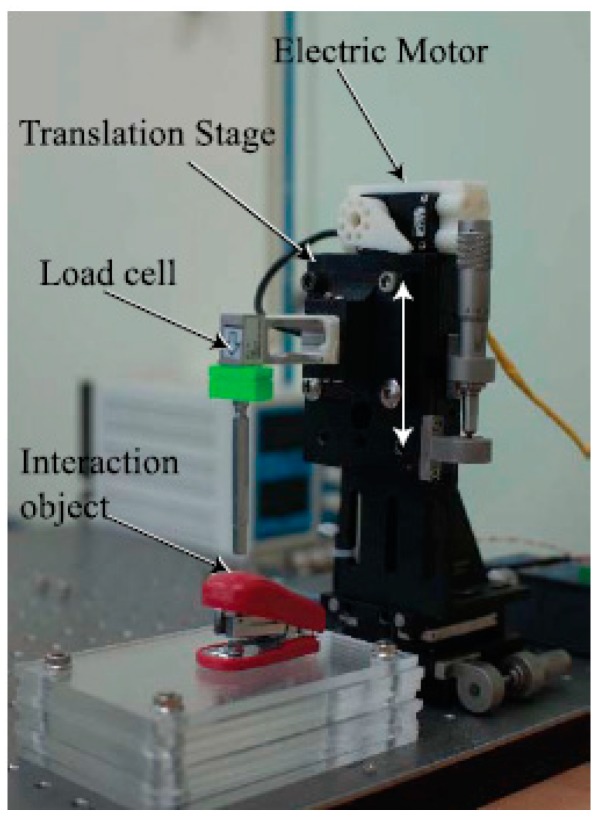
Interaction force measurement system with load cell and translation stage with electric motor.

**Figure 8:**
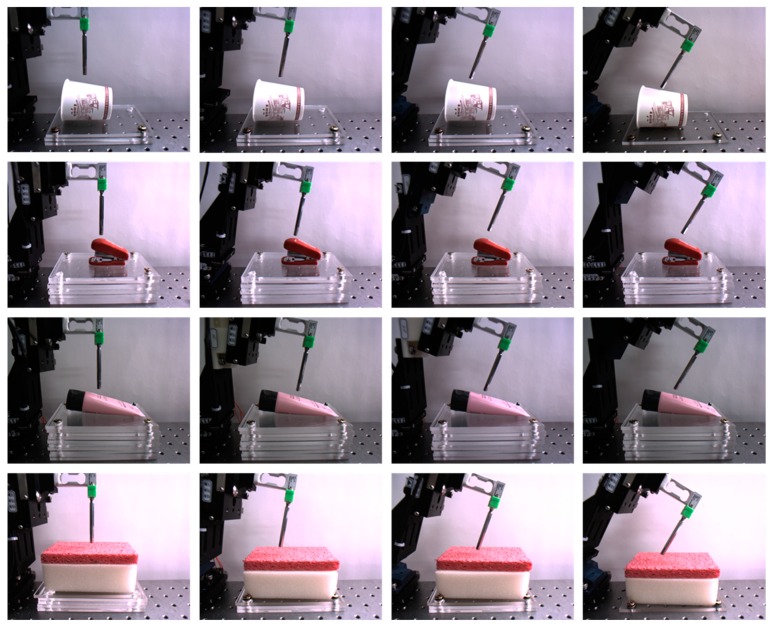
Collected sample images. From the top to bottom rows, paper cup, stapler, tube, and sponge are shown; angle variations are from the left to right columns. The first and third-row images were captured with 350-lux illumination, whereas the second and fourth-row images were taken with 550 and 750-lux illuminations, respectively.

In this study, a new database collection system was designed, as shown in Figure 7. The system was equipped with a long steel rod to enable point touch with the target object. On top of the rod, a load cell (model BCL-1L, CAS) was installed to measure the interaction force between the rod and target object. In [13], the translation stage movement was controlled by the operator; however, in the present study, the movement of the translation stage was driven by the RC servo-motor. This motor could automatically move the translation stage along a single direction (for example, *z*-direction); moreover, randomized magnitudes of the external force and the period of touch could be implemented through the motor control code. In this respect, the main goal of this study was to predict the unknown and random interaction forces by means of the deep learning method free of human intervention. For a more varied experimental environment, different illumination conditions, which typically affect camera vision, and different angles for the translation stage movement were implemented. Three different light conditions (350, 550, and 750 lux) were employed, and four different angles (0°, 10°, 20°, and 30°) were designed for the movement. The sequential images were captured using a 149-Hz camera (Cameleon3, CM3-U3-13Y3C-CS, Pointgrey) positioned in front of the collecting system. Apart from capturing images, the interaction force that corresponded to each image was measured and recorded using the loadcell. To synchronize the force and image, the camera device provided a time stamp. Four different target objects (paper cup, stapler, tube, and sponge) were employed, as shown in Figure 8; the paper cup and sponge have rich textures, whereas those of the others (stapler and tube) are flat. In terms of deformation, because the stapler has two different parts that are connected by a hinge, the external force causes it to deform differently when compared with the other three objects.

### 4.2. Evaluation Protocol

To build the training and test protocols, a total of 387,473 sequential images were captured using an RGB camera, and the corresponding interaction forces were measured by the load cell. Compared with [13], the number of images in the new database was six times larger. The collected image resolution was 1280 × 1024 (RGB) pixels, and its corresponding interaction force was a single value. One set consisted of four contacts to the target object, and a total of 15 sets were collected from each environment. Accordingly, approximately 380,000 sequential images (15 sets × 520 images × 4 objects × 3 light conditions × 4 angles) were collected (The database will be released at https://github.com/hyeon-jo/Interaction-force-estimation-based-on-deep-learning). The database was divided into training and test sets, as summarized in Table 3; there were 309,853 and 77,620 training and test images, respectively. The deep learning model was trained starting from zero. Note that the maximum external forces were generated by the random function with the main goal of training the deep learning model to be capable of inferring the unknown forces only from images without the aid of any haptic sensor. The performance between the ground truth (GT) and predicted force was measured using the root mean square error (RMSE).

### 4.3. Implementation Details

**Figure 9:**
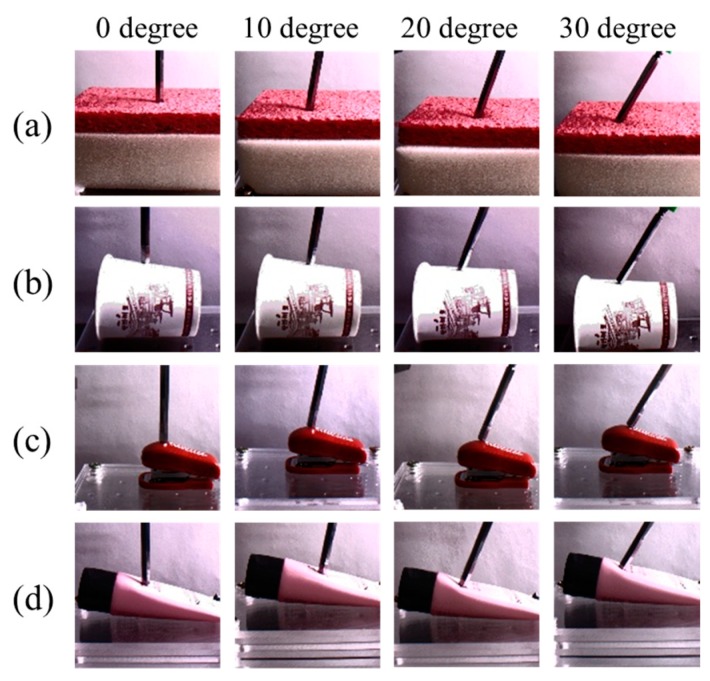
Cropped and normalized sample images. (**a**) Sponge, (**b**) paper cup, (**c**) stapler, and (**d**) tube.

The input images were cropped around the touching area where the target object and instrument came into contact, as shown in Figure 9; this is because more information could be obtained by sensing the movement of the transition stage rather than the deformation of the target object by an external force. The cropped images were normalized into 128 × 128 × 3 (RGB) pixel-based images; details of the network architecture are summarized in Table 2. In the training stage, a mini-batch with 128 samples and 20 sequential training frames was created. Initially, the learning rate was 0.001; after 750 iterations, it gradually decreased. The model was trained through 1,500,000 iterations (approximately 100 epochs). The learning rate at the ith iteration was calculated by the following equation:

(2) $$LR_{i}=0.001\times 0.9^{i/750}$$

**Figure 10:**
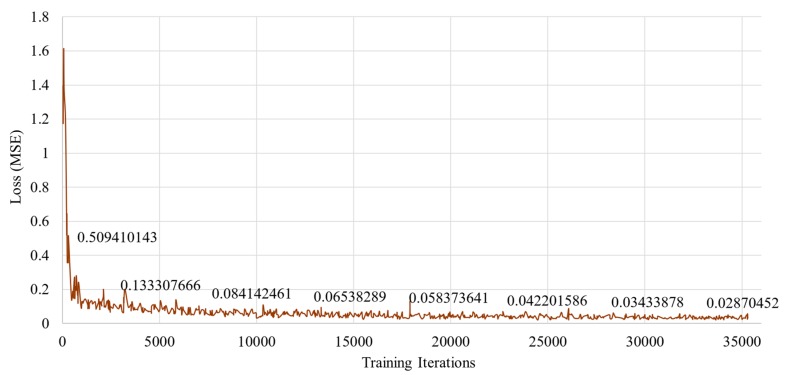
Training loss in the proposed method for predicting interaction forces from images only.

The loss function is the mean square error between the measured force ($y_{i}$) by the load cell, and the estimated force ($\hat{y_{i}}$) by the proposed method is given by the following equation:

(3) $$MSE\left(y_{i},\hat{y_{i}}\right)=\frac{1}{n}\sum \left(y_{i}-\hat{y_{i}}\right)$$

where n is the number of samples. The Xavier initialization [31] was used for weight initialization, and the Adam Optimizer [32] was used to optimize the neural network parameters. The model was trained starting from zero. As shown in Figure 10, the proposed method could be successfully trained without any training tricks (e.g., fine-tuning with a pre-trained model) although it is designed for an optimized and lightweight network architecture. The proposed method was implemented by Tensorflow, and all experiments were performed with four Nvidia Titan V GPUs.

## 5. Experimental Results and Discussion

In this section, the performance of the proposed method is presented by comparing it with well-known methods such as the CNN + LSTM [22], the 3D CNN-based method, and the 2D CNN-based CNN method [25]. This is because our key contribution is the introduction of the lightweight 3D CNN method to infer the interaction force only from sequential images, and the natural baseline is the CNN + LSTM, which is a widely adopted method for exploiting the temporal information in computer vision applications. For a fair comparison, all previous works were trained under the same protocol listed in Table 3. The quantitative results of the overall performances in the prediction of the interaction forces by the neural network are presented first. The measurement accuracy was calculated by the mean of the average RMSE (the average RMSE is the average RMSE of all frames of a target object, and the mean of the average RMSE is the mean of the four averages of the RMSE, i.e., that of the sponge, paper cup, stapler, and tube).

### 5.1. Overall Performances

**Figure 11:**
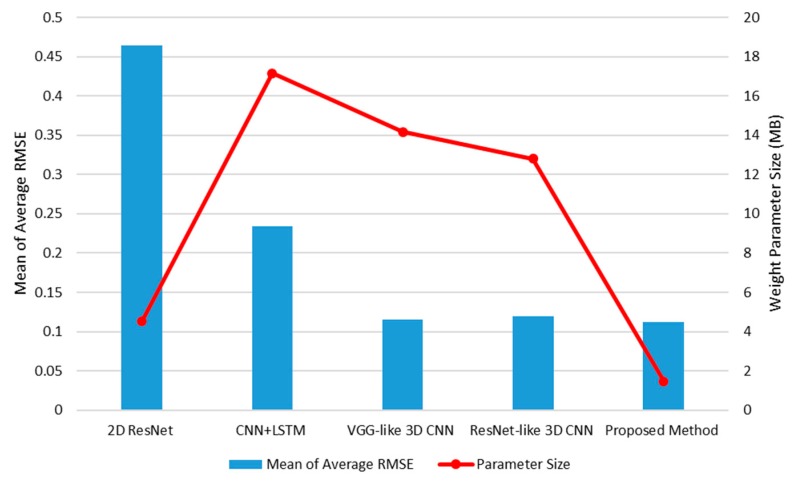
Overall performances of the 2D CNN-based ResNet with 18 layers, the CNN + LSTM, the traditional 3D CNN-based methods (i.e., 18 layer-based VGG style method and 19 layer-based ResNet style method), and the proposed method.

For a fair evaluation, four different methods (i.e., the 2D CNN-based ResNet, the CNN + LSTM, the VGG-like 3D CNN method, and the ResNet-like 3D CNN method) and the proposed method were implemented. As shown in Figure 11, the first method was the 2D CNN-based ResNet with 18 layers. Note that this method only uses a single input image, whereas the other methods use 20 frames as input for the CNN + LSTM and 3D CNN. The parameter size of the 2D CNN-based ResNet is only 4.54 MB, which is not large. Unlike the other methods, the 2D CNN-based ResNet does not utilize temporal information. That is, the texture and shape information in a single image were analyzed to predict the interaction force. As a result, the final accuracy was the lowest when compared with the others. The CNN + LSTM was implemented only for visual information without electronic information; the input images were 20 sequential frames. The parameter size was the largest (i.e., 17.16 MB) because its main function is not to build a compact network architecture, but to achieve good force estimation accuracy. However, the final accuracy was also not better than that of the 3D CNN-based methods. Accordingly, it can be concluded that in terms of performance, the basic CNN + LSTM architecture was not as effective as the 3D CNN-based method.

The 3D CNN-based methods were also examined in terms of inference accuracy and weight parameter size. Two different 3D CNN-based methods (18 layer-based VGG style 3D CNN method and 19 layer-based ResNet style 3D CNN method) were implemented in this study. These methods differed from the 2D-based VGG and ResNet methods only in terms of convolutional kernels (3D for the former and 2d for the latter). Similar to the CNN + LSTM method, the inputs for the 3D CNN-based methods were also 20 sequential frames. Another difference between the VGG style 3D CNN and ResNet style 3D CNN was the presence of the residual short connection proposed in ResNet [25]. In terms of inference accuracy, two different 3D CNN methods exhibited similar average RMSE means (i.e., 0.1157 and 0.1193 for the VGG and ResNet style methods, respectively); their parameter sizes were also similar (i.e., 14.16 and 12.80 MB, respectively). Although the VGG style method employs two not fully connected layers at the higher layers and the global average pooling, its parameter size was not larger than expected. The VGG style 3D CNN method was more accurate and larger in parameter size than the ResNet style 3D CNN method because the former had one more layer than the latter. Finally, when compared with these two methods, the proposed method had the smallest parameter size (i.e., 1.47 MB) and best accuracy (i.e., 0.1120 mean of average RMSE). The proposed method also had the same number of input frames as the other methods; compared with other 3D CNN methods, approximately 10 times less parameter size and better accuracy were obtained. This overall performance comparison concludes that the proposed 3D CNN lightweight method is superior in terms of accuracy and parameter size.

### 5.2. Investigation of Different Material-Based Objects

**Figure 12:**
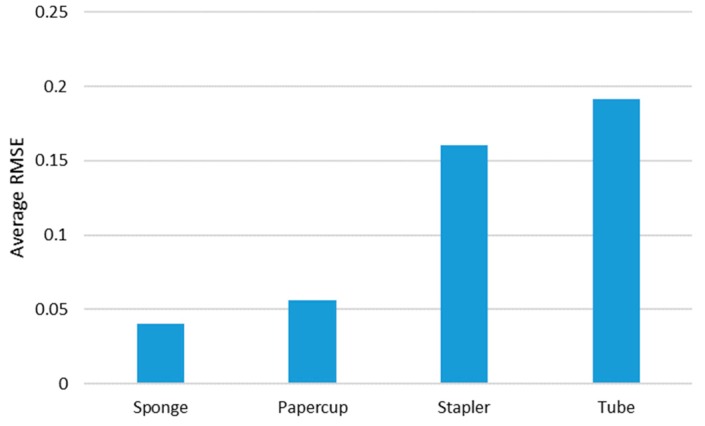
Average RMSE of the proposed method with each tested object.

This section presents the investigation of the performance of the proposed method with each target object (sponge, paper cup, stapler, and tube). In Figure 12, the performances of the proposed method with the target objects are shown in ascending order as follows: sponge, paper cup, stapler, and tube. Based on the average RMSE, two groups could be identified: a relatively high accuracy was achieved by one group (i.e., sponge and paper cup) and the other group (stapler and tube) had a relatively low accuracy; the difference between these two accuracies was approximately three times the lower accuracy. However, the performance of the pair with low accuracies with the proposed method was better than that when the CNN + LSTM (mean of average RMSE: 0.2341) was used. There may be several intrinsic and extrinsic reasons for this difference; however, it is important to determine whether or not the structure of the target object is complex. As shown in Figure 8, the sponge and paper cup have simple structures, and their surfaces contain rich texture information. Accordingly, the changes in their shapes caused by external forces were linear, and the changes in visual information were clearly observed because of this texture. On the other hand, the other objects were more complex: the stapler has two parts joined by a hinge, and the tube consists of a solid plastic cap and a soft body; their surfaces do not have rich textures. Nevertheless, it should be emphasized that the performance difference among these objects is relative; their actual performances with the existing 3D CNN-based method was similar to or better than those with the CNN + LSTM and 2D CNN-based methods. In the subsequent section, the details of individual material properties are presented through quantitative comparisons.

#### 5.2.1. Sponge

**Figure 13:**
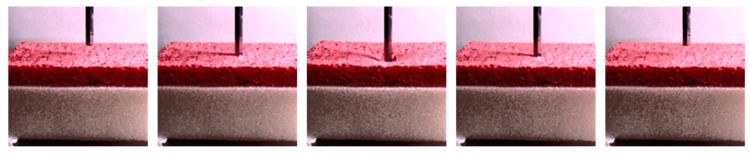
Sample images of sponge.

**Figure 14:**
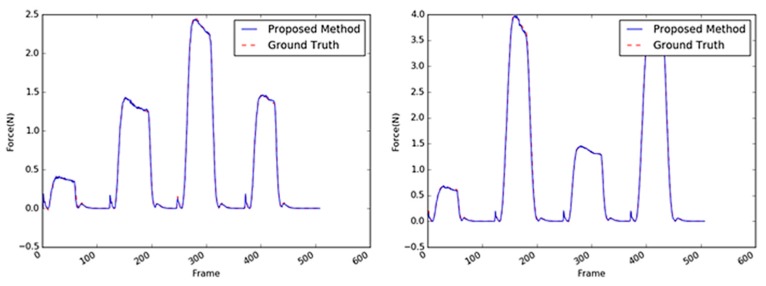
Inferred interaction forces of the sponge with the GT. The blue and red dotted lines represent the results of the proposed method and the GT measured by the load cell, respectively. Maximum external forces are randomly generated.

Sample images of the sponge (the first target object) are shown in Figure 13. A sponge is an ideal target object because it is not difficult to predict its interaction force from the video input. This is mainly because of the regular hole patterns that are abundant on the sponge surface; hence, the interaction force can be modeled linearly. Even when the external force is applied to any point of the sponge, the tensile force in the material remains considerable, and the change in a large area varies with the texture. The comparison of the performances of the three methods with the sponge as the target object is summarized in Table 4. With the sponge, the performance of the 2D ResNet [25] exhibited the least accuracy; the performance of the proposed method was four times better or more. The performance of the proposed method with the sponge as target was more than twice the performance of the CNN + LSTM method; it was also similar or slightly better than the performances of the other 3D CNN-based methods. Some results are shown in Figure 14 where the estimated results were practically the same as the GT values. Note that the magnitudes of the maximum forces and the period between the application of two consecutive forces were randomly generated. In this respect, it can be concluded that the proposed method achieves an acceptable accuracy despite having a parameter size that is 10 times smaller than the other methods.

#### 5.2.2. Paper Cup

**Figure 15:**
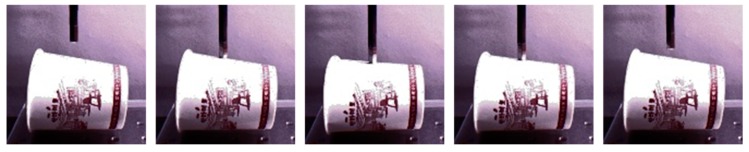
Sample images of the paper cup.

**Figure 16:**
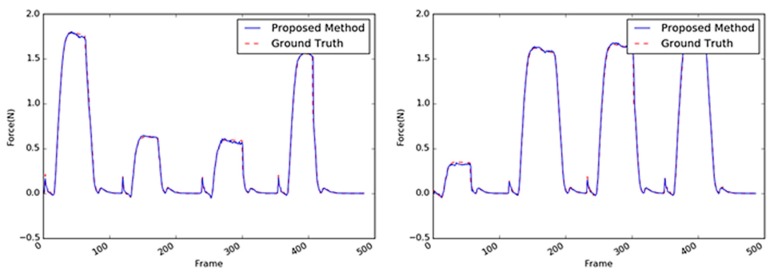
Inferred interaction forces for the paper cup with the GT. The blue and red dotted lines represent the results of the proposed method and the GT measured by the load cell, respectively. Note that the durations of the maximum force applied were randomly generated.

Another target object was a cup, whose core material was a coated paper, as shown in Figure 15. The performance comparison results of the various methods with paper cup as the target object are summarized in Table 5; the results exhibit a similar tendency as those with the sponge as the target object. The performance of the proposed method with the paper cup achieved the best accuracy with an average RMSE of 0.0560; the differences of this performance from those obtained using the other algorithms were similar to those obtained from the sponge experiments except for the absolute values of the average RMSE that ranged 0.0405–0.0560. Overall, with the paper cup as the target object, the 3D CNN-based methods performed better than the 2D CNN-based methods. In Figure 16, it can be observed that the proposed method yielded practically the same results as the GT, although the weight parameters were reduced. The vision-based force estimation performed well with the paper cup because this material has sufficient textural information on its surface, particularly the side facing the camera. Moreover, when the top of the paper cup was pressed, large areas in the object considerably deformed in light of the paper’s characteristics. Sequential images facilitated the detection of changes in the object and the measurement of the external force with the variation. Accordingly, it was confirmed that the proposed method could produce satisfactory results using only the visual information from the paper cup.

#### 5.2.3. Stapler

**Figure 17:**
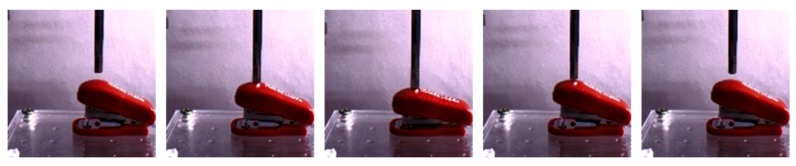
Sample images of the stapler.

**Figure 18:**
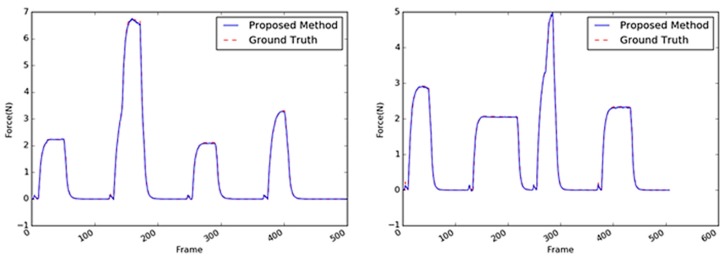
Inferred interaction forces of the stapler with the GT. The blue and red dotted lines represent the results of the proposed method and the GT measured by the load cell, respectively.

The stapler, shown in Figure 17, is a type of complex structure. Two different rigid parts are combined with a hinge, and a spring supports the two parts between the hinge. When an external force acts on the upper part of the stapler, the interaction force measured by the load cell slightly differed from that of the previous objects because of the spring’s repulsive force. As can be observed in Figure 18, when a strong force was applied, the graph of the interaction force showed a slightly different pattern upon reaching a certain point (i.e., 3–4 N). Moreover, the stapler surface does not possess sufficient texture information, so its deformation was not distinct. Thus, the average RMS of the stapler was three to four times worse than the two previous objects, as indicated in Table 6. The best accuracy (average RMSE: 0.1554) was achieved by the VGG-like 3D CNN-based method; however, the average RMSE of the proposed method (i.e., 0.1602) was practically like that of the former. However, the proposed method exhibited better accuracy by approximately two times that of the 2D CNN-based methods such as the CNN + LSTM and 2D CNN-based ResNet methods.

#### 5.2.4. Tube

**Figure 19:**
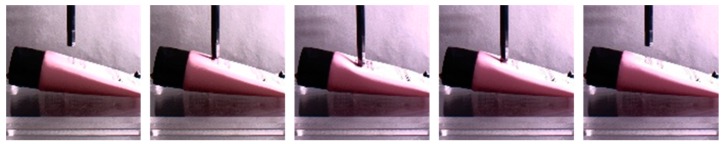
Sample images of the tube.

**Figure 20:**
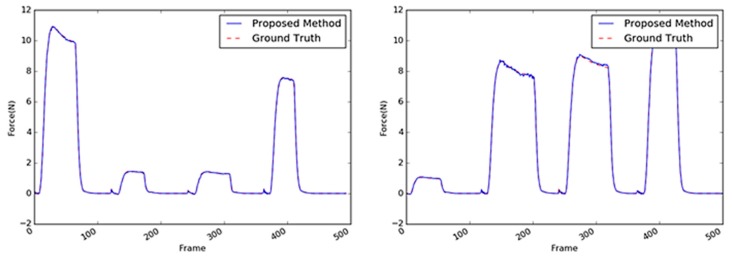
Inferred interaction forces of the tube with the GT. The blue and red dotted lines represent the results of the proposed method and the GT measured by the load cell, respectively.

The sample images of the tube are shown in Figure 19. The performances of the methods with the tube as the target object exhibited the relatively worst accuracies when compared with the performances of these methods with the other objects as the target. This indicates that it is difficult to infer the interaction force with the tube using only visual information. Based on the average RMSE, there was a drop in the performance of the CNN + LSTM method with the stapler as the target object (0.3385 in Table 6) and the tube as the target (0.4046 in Table 7). On the other hand, the 3D CNN-based methods achieved better accuracies (0.2075 and 0.1345), as listed in Table 7. The structure of the tube is also complex; its cap is rigid, whereas its body is made of soft material. The contact area was set to be on the soft material of the tube, which does not have sufficient textural information; moreover, its deformation was not apparent when compared with those of the other objects. As shown in Figure 20, the tube’s peak area characteristics resulting from the external maximum forces were similar to those of the sponge. Figure 20 indicates that there were a few differences between the estimated maximum values and the GT.

### 5.3. Discussion

**Figure 21:**
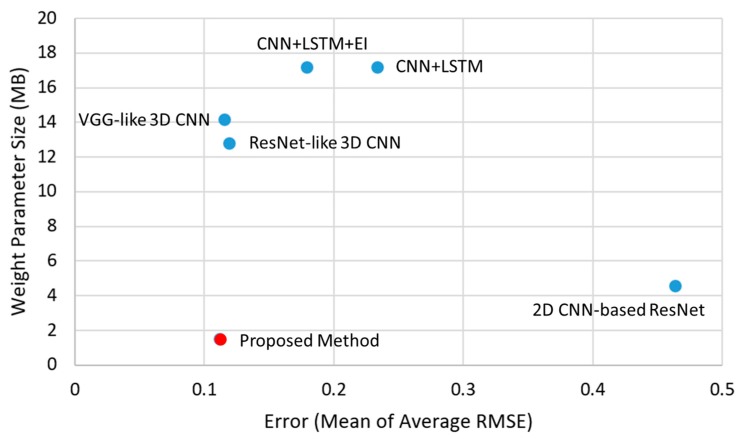
The proposed method achieved the best accuracy in inferring the interaction force only from the video; its weight parameter size was also the smallest among the previous works.

To compare the characteristics of the various network architectures in detail, Figure 21 presents the overall performances of the following networks: the 2D CNN, CNN + LSTM, 3D CNN variants, and the proposed method. In terms of estimation accuracy, the highest-ranking methods were the proposed method and the two (VGG-like and ResNet-like) 3D CNN-based methods. From the viewpoint of only accuracy, the 3D CNN-based methods always achieved better performances than the others. Moreover, the proposed method had approximately 6–7 times smaller weight parameter sizes than the 3D CNN variant methods. The models with smaller weight parameter sizes and the 2D CNN are the preferred methods. However, the proposed method is approximately four times better in terms of accuracy than the 2D CNN. Apart from the foregoing, the proposed method exhibited superiority in terms of parameter size and accuracy than the 2D CNN + LSTM method, which is generally used for deep learning-based video processing. All in all, the proposed method results in good accuracy with the light-weight parameters.

**Figure 22:**
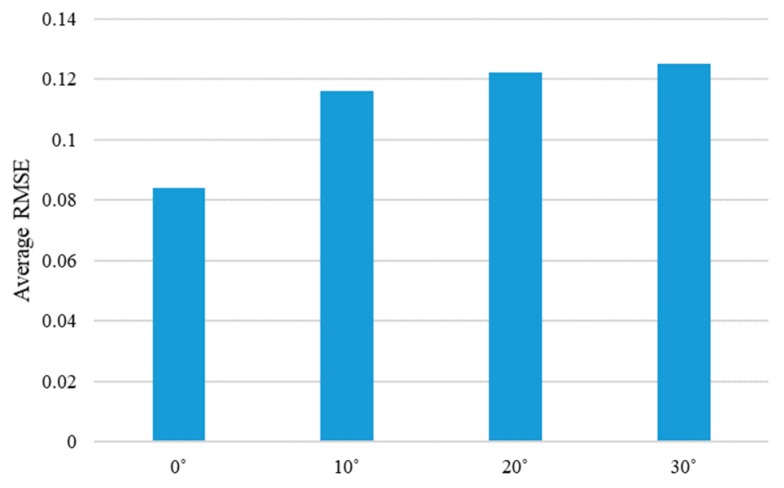
Average RMSE of the proposed method are shown according to the different moving directions of the steel rod. Average RMSE is calculated by averaging the RMSE values for each angle of the four objects.

**Figure 23:**
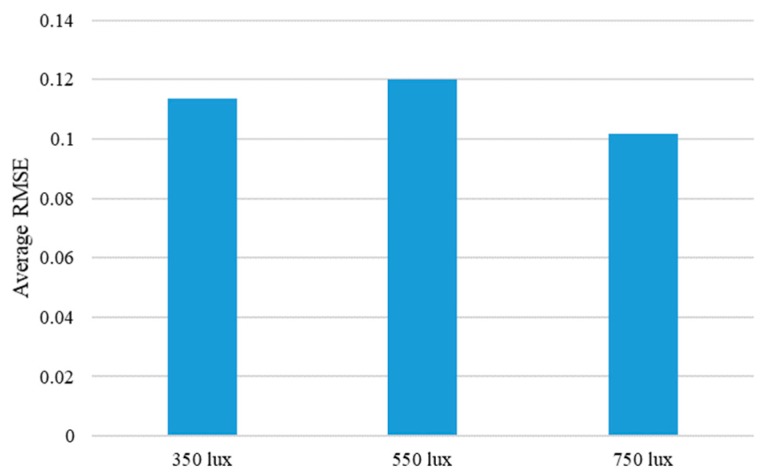
Average RMSE of the proposed method are shown according to the different illumination conditions. Average RMSE is calculated by averaging the RMSE values for each illumination condition of the four objects.

To further analyze the performance of the proposed method, the average RMSE values, according to the different experiment environments of four objects, was calculated. The performance change according to the moving direction of the steel rod showed the best performance at 0 degrees, and the performance worsened as the angle increased, as shown in Figure 22. This is largely because the moving distance of the steel rod is always constant regardless of the angle, so the larger the entry angle of the rod, the smaller the moving distance in the y-axis direction. Therefore, the texture or shape change of the object becomes smaller. Another experimental condition, the performance, changes according to the different illumination conditions, is shown in Figure 23. As expected, the best accuracy was achieved by the 750 lux condition. Although the performance order was different from the expected one at 350 lux and 550 lux, it showed almost similar average RMSE values. In detail, the difference of the average RMSE values between 350 lux and 550 lux was only 0.0065, while the difference between 350 lux and 750 lux was 0.0118. From these two figures, it can be seen that the proposed algorithm was not significantly affected by the lighting condition, and the change in the entry angle of the steel rod had a relatively large influence on the performance of inferring the interaction force from the video.

**Figure 24:**
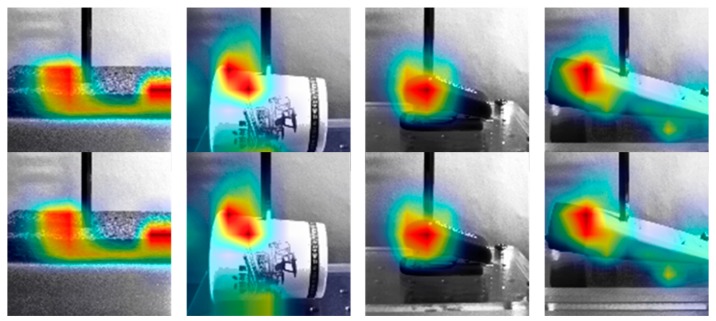
The attention maps of the proposed method are shown according to the different materials.

The attention mechanism [33] is a well-known method for visualizing the important parts in the deep learning network. For the visualization of the force inference by the proposed method, we added the simple attention module to the proposed network architecture. Figure 24 shows the results of the attention module according to the different materials, which shows where the image appearances largely changed over time in the video. From these results, we can conclude that the proposed method, whose main purpose is inferring the interaction forces from the video, makes use of the texture changes according to the external force applied.

## 6. Conclusions and Future Works

In this paper, a 3D depthwise separable convolution neural network was proposed for inferring the interaction force from video input. Among the 3D CNN-based methods with small weight parameters, the proposed method exhibited better performances than those reported in previous works. Based on the experimental results, it was confirmed that the proposed method was successfully modified to the 3D CNN-based method, and the parameter size of its convolutional filters was reduced by 10 times without causing degradation in its accuracy. To prove our hypothesis, we collected a considerable quantity of videos and their corresponding interaction forces using the automatic database collection system. The results of this experiment confirmed that the inference of abstract information such as a physical phenomenon can be reproduced by a convolution-based neural network; this can be achieved by recognizing and understanding the context of the surrounding visual information as humans do. Moreover, even with limited resources, the proposed method can be utilized for developing robots with good recognition capacities. In the near future, we will collect other different objects in the database. Moreover, we will develop another experimental protocol where we will learn the deep learning models by subtracting some variations and checking whether the learned model corresponds well to the variations not included in the training stage.

## Figures and Tables

**Table 1:** Comparison of weight parameters between 3D CNN and proposed method.

	3D CNN	Proposed Method
No. of Parameters	n×c×d×(k×k+1)	n×(c×d+1)+c×(k×k+1)

**Table 2:** Network architecture details of the proposed method.

Input Size <d, h,w,c>	Layer (Type)	Expand Channels	Output Channels	Spatial Stride	Kernel Depth	Depth Stride
<20, 128, 128, 3>	Conv2D 3 × 3	-	32	1	1	1
<20, 64, 64, 32>	Bottleneck 3D 3 × 3 (a)	32	16	1	1	1
<20, 64, 64, 16>	Bottleneck 3D 3 × 3 (a)	64	24	1	1	1
<20, 32, 32, 24>	Bottleneck 3D 3 × 3 (a)	96	32	1	1	1
<20, 16, 16, 32>	Bottleneck 3D 3 × 3 (b)	128	64	2	3	2
<10, 8, 8, 64>	Bottleneck 3D 3 × 3 (b)	192	92	2	3	2
<5, 8, 8, 92>	Bottleneck 3D 3 × 3 (b)	384	128	2	3	2
<3, 4, 4, 128>	Bottleneck 3D 3 × 3 (b)	448	192	2	3	2
<2, 4, 4, 192>	Conv2D 1 × 1	-	1280	2	2	2
<1, 4, 4, 1280>	Avg. Pool. 4 × 4	-	-	1	1	-
<1280>	FC 1	-	1	-	-	-

**Table 3:** Details of training and test protocols. One set consists of four touches with approximately 520 sequential images. The number in parentheses is the total number of sequential images. The total number of images is 387,473.

Object	Training Set	Test Set
Sponge	144 sets (77,097)	36 sets (19,474)
Paper cup	144 sets (76,966)	36 sets (19,133)
Stapler	144 sets (77,941)	36 sets (19,533)
Tube	144 sets (77,849)	36 sets (19,480)

**Table 4:** Quantitative comparison of various methods with the sponge as the target object.

Method		Average RMSE
2D CNN-based methods	2D CNN-based ResNet [25]	0.1844
	CNN + LSTM	0.0925
	CNN + LSTM + EI [21]	0.0882
3D CNN-based methods	VGG-like 3D CNN	0.0422
	ResNet-like 3D CNN	0.0757
	Proposed Method	0.0405

**Table 5:** Quantitative comparison of various methods with the paper cup as the target object.

Method		Average RMSE
2D CNN-based methods	2D CNN-based ResNet [25]	0.2049
	CNN + LSTM	0.1007
	CNN + LSTM + EI [21]	-
3D CNN-based methods	VGG-like 3D CNN	0.0580
	ResNet-like 3D CNN	0.0737
	Proposed Method	0.0560

**Table 6:** Quantitative comparison of various methods with the stapler as the target object.

Method		Average RMSE
2D CNN-based methods	2D CNN-based ResNet [25]	0.7526
	CNN + LSTM	0.3385
	CNN + LSTM + EI [21]	0.2702
3D CNN-based methods	VGG-like 3D CNN	0.1554
	ResNet-like 3D CNN	0.1933
	Proposed Method	0.1602

**Table 7:** Quantitative comparison of performances of various methods with the tube as the target object.

Method		Average RMSE
2D CNN-based methods	2D CNN-based ResNet [25]	0.7149
	CNN + LSTM	0.4046
	CNN + LSTM + EI [21]	-
3D CNN-based methods	VGG-like 3D CNN	0.2075
	ResNet-like 3D CNN	0.1345
	Proposed Method	0.1914
